# Celebrating 350 years of *Philosophical Transactions*: physical sciences papers

**DOI:** 10.1098/rsta.2014.0472

**Published:** 2015-04-13

**Authors:** Dave Garner

**Affiliations:** Editor-in-Chief

This theme issue of *Philosophical Transactions A* has been produced as an integral component of the Royal Society's celebrations of the 350th anniversary of the publication of the first issue of *Philosophical* *Transactions*, the world's oldest scientific journal. The issue contains 16 commentaries, each of which is focused on a major scientific advance in the physical, mathematical or engineering sciences since 1665 that was published in *Philosophical Transactions*. A corresponding theme issue of the journal's biological sciences counterpart, *Philosophical Transactions B*, has also been produced [1].

The Royal Society booklet ‘*Philosophical Transactions*: 350 years of publishing at the Royal Society (1665–2015)’ [2] provides a fascinating account of the origins of *Philosophical Transactions* and its evolution, together with some biographical details of the key individuals involved. On 6 March 1665, the first issue of *Philosophical Transactions* was published under the visionary editorship of Henry Oldenburg, who was also the Secretary of the Society. Oldenburg was born and educated in Bremen and then in Utrecht and Oxford. By *ca* 1660 Oldenburg was settled in London, following a varied career which included tutoring sons of English noblemen and being engaged in diplomatic missions as Bremen's envoy to Oliver Cromwell. The early issues of the journal were compiled by Oldenburg and consisted of edited aspects of his correspondence, reviews and summaries of recently published books, together with accounts of observations and experiments from natural philosophers based in the UK and mainland Europe. The information presented in Volume 1 [3] is fascinating. The text commences with a ‘Dedicatory Epistle’ to the Royal Society by Oldenburg that concludes with the solicitations: *The Great God prosper You in the Noble Engagement of Dispersing the true Lustre of his Glorious Works, and the Happy Inventions of obliging Men all over the World, to the General Benefit of All Mankind*. This volume contains a wide range of correspondence on topics as scientifically diverse as:

*A Spot in One of the Belts of Jupiter*; *An Account of Micrographia, or the Physiological Descriptions of Minute Bodies, Made by Magnifying Glasses*; and *Of the Mineral of Liege, Yielding Both Brimstone and Vitriol, and the Way of Extracting Them Out of It, Used at Liege*. Thus, the wide scientific range of the journal was established in this first issue.

**Figure 1.**
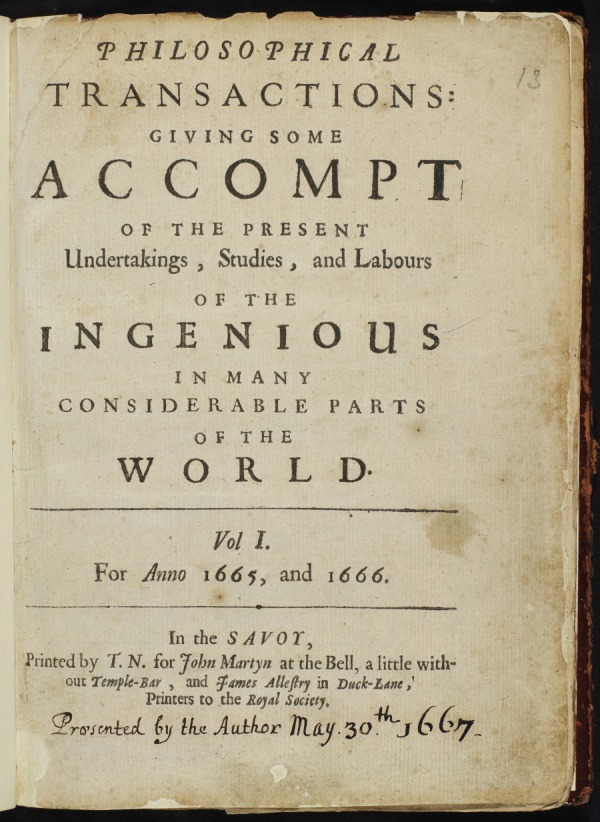
A picture of the title page of Volume 1.

The original title of the journal was *Philosophical Transactions Giving Some Account of the Present Understanding, Studies and Labours of the Ingenious in Many Considerable Parts of the World* (figure 1). Although the first volumes had a longer title and were very different in style from today's journal, in essence they served the same function, i.e. to inform the Fellows of the Society and other interested readers of the latest scientific discoveries and developments. Furthermore, from the beginning, the journal helped to establish the two important principles of scientific priority and peer review, which have become the central foundations of all of the leading scientific journals. At the outset, the journal focused on the latest scientific discoveries and developments, and often reported on current, rather than completed, research. Accurate recording of empirical observations and experimental measurements was paramount and regarded as the route to understanding the true nature of things. The majority of the contributions were in English although some—notably astronomical and mathematical—papers were in Latin.

In its early years, the production of *Philosophical Transactions* suffered major interruptions due to the Plague of 1665 and the Great Fire of London in 1666. Although publication of the first few issues commenced in the heart of Old London town, near St Paul's, it was forced to move to Oxford to escape the Plague. Then, no sooner had the Plague been subdued by a particularly harsh winter, making it safe to return to London, when publication was again interrupted by the Great Fire. Then, in 1667, Henry Oldenburg was briefly imprisoned in the Tower of London on suspicion of passing information to the enemy during the second Anglo-Dutch war (1665–1667); this arose due to his scientific correspondence with a Dutch colleague.

Following Oldenburg's death in 1677, the responsibility of editing *Philosophical Transactions* was undertaken by a series of scientists, the majority of whom were the serving Secretary of the Royal Society. This required a considerable devotion to the cause since the Editor-Secretary bore the financial burden of publishing the journal. The Royal Society's reputation became increasingly linked to that of *Transactions*. During the period 1720–1750, the society and the journal both experienced biting satirical attacks from writers such as Jonathan Swift (e.g. *Gulliver's Travels*) and the actor, apothecary and naturalist John Hill. This ridicule led to the journal being brought under the direct control of the Royal Society in 1752 and renamed as the *Philosophical Transactions of the Royal Society* in 1786. Further reform followed in the nineteenth century, when Charles Babbage wrote an open letter, denouncing Fellows who used the Royal Society as a social club and calling for a new category of Fellow: working scientists who published their work in *Philosophical Transactions*. By the late 1830s, peer review was made more rigorous and (usually) involved two or more referees, one of which could be the Editor, a position then still held by the Secretary of the Society.

By the late nineteenth century, the breadth and scope of scientific discovery had increased to such an extent that (in 1886) it was decided to divide the journal into two components, *Philosophical Transactions A*, covering the physical sciences, and *Philosophical Transactions B*, covering the biological sciences. In the later twentieth century much of the administrative and marketing responsibilities for the journals were taken on by in-house publishing editors, with a Fellow at the helm of each journal supported by a large, internationally and scientifically diverse editorial board.

In 1996, given significant competition from specialist journals, the decision was made that *Philosophical Transactions* would adopt a unique publishing style. Each issue would focus on a specific topic of current scientific importance and be guest-edited by one (or more) leading scientist(s) in the field. Each year, several issues of both *Philosophical Transactions* *A* and *B* are based on the presentations given at a Royal Society Discussion or Theo Murphy meeting. This approach has proved to be successful and, currently, 26 issues of both *Philosophical Transactions* *A* and *B* are published annually.

Given that *Philosophical Transactions* has published a significant number of the most important scientific discoveries and developments accomplished since 1665, the selection of the landmark papers in the physical, mathematical or engineering sciences to be celebrated in this theme issue represented a considerable challenge. The process commenced with the consideration of over 300 possible publications from which a ‘long list’ of *ca* 70 papers was produced. These papers were then carefully reviewed and subjected to the following considerations:

(i) the paper must have had a high scientific impact when published and remain influential today;
(ii) the need to ensure a balanced representation of the disciplines that *Philosophical Transactions A* currently covers;
(iii) the desirability of including contributions from all of the various time periods across the journal's 350 year history; and
(iv) due to the considerable competition and the limitations of space, no scientist could be represented by more than one publication.

The 16 landmark papers selected received the approval of the journal's Editorial Board. The fact that only two of these papers celebrate the achievements of female scientists is a sad reflection of the historical under representation of women in the world of science.

Each landmark paper is celebrated by a commentary that places the scientific advance in its historical and scientific context and discusses how the new knowledge obtained has influenced developments within its scientific field, together with considerations of the wider impact of the discovery. I greatly enjoyed reading these accounts and I hope that that they will provide readers of this theme issue of *Philosophical Transactions A* with an insight into the essence of each of the major scientific developments, a perspective of their significance and an appreciation of some of the pertinent human dimensions.

This theme issue of *Philosophical Transactions A* illustrates the major contributions to the development of scientific knowledge and understanding made by the journal during its lifetime. In addition to celebrating past achievements, we look forward to the journal continuing to play a significant role in providing authoritative and readily accessible accounts of recent notable scientific achievements, together with developments in prospect and challenges beyond our present horizons.
